# Evaluation of Facebook as a Longitudinal Data Source for Parkinson’s Disease Insights

**DOI:** 10.3390/jcm14124093

**Published:** 2025-06-10

**Authors:** Jeanne M. Powell, Charles Cao, Kayla Means, Sahithi Lakamana, Abeed Sarker, J. Lucas Mckay

**Affiliations:** 1 Department of Biomedical Informatics, Emory School of Medicine, Atlanta, GA 30030, USA; sahithi.krishnaveni.lakamana@emory.edu (S.L.); abeed.sarker@emory.edu (A.S.); j.lucas.mckay@emory.edu (J.L.M.); 2 Rollins School of Public Health, Emory University, Atlanta, GA 30030, USA; charles.cao@emory.edu (C.C.); kayla.means@emory.edu (K.M.)

**Keywords:** Parkinson’s disease, social media, text classification

## Abstract

**Background/Objectives:** Parkinson’s disease (PD) is a neurodegenerative disorder with a prolonged prodromal phase and progressive symptom burden. Traditional monitoring relies on clinical visits post-diagnosis, limiting the ability to capture early symptoms and real-world disease progression outside structured assessments. Social media provides an alternative source of longitudinal, patient-driven data, offering an opportunity to analyze both pre-diagnostic experiences and later disease manifestations. This study evaluates the feasibility of using Facebook to analyze PD-related discourse and disease timelines. **Methods**: Participants (N = 60) diagnosed with PD, essential tremor, or atypical parkinsonism, along with caregivers, were recruited. Demographic and clinical data were collected during structured interviews. Participants with Facebook accounts shared their account data. PD-related posts were identified using a Naïve Bayes classifier (recall: 0.86, 95% CI: 0.84–0.88, AUC = 0.94) trained on a ground-truth dataset of 6750 manually labeled posts. **Results**: Among participants with PD (PwPD), Facebook users were significantly younger but had similar Movement Disorder Society-United Parkinson’s Disease Rating Scale scores and disease duration compared to non-users. Among Facebook users with PD, 90% had accounts before diagnosis, enabling retrospective analysis of pre-diagnostic content. PwPD maintained 14 ± 3 years of Facebook history, including 5 ± 6 years pre-diagnosis. On average, 3.6% of all posts shared by PwPD were PD-related, and 1.7% of all posts shared before diagnosis were PD-related. Overall, 69% explicitly referenced PD, and 93% posted about PD-related themes. **Conclusions**: Facebook is a viable platform for studying PD progression, capturing both early content from the premorbid period and later-stage symptoms. These findings support its potential for disease monitoring at scale.

## 1. Introduction

Social media platforms are used by nearly 60% of the global population [1], with Facebook alone reporting 2.89 billion active users in 2024 [2]. Beyond maintaining social connections [3], older adults use social media to share aspects of their daily lives, including health-related experiences [4,5,6]. These posts offer rich, real-world data that can be mined for medical insight. However, given their unstructured and often informal nature, analyzing large volumes of social media content requires computational tools.

Natural language processing (NLP) is a branch of artificial intelligence that enables machines to analyze and interpret human language. In healthcare, NLP has emerged as a powerful method for transforming unstructured text—such as clinical notes [7] or social media posts [6,8]—into structured data. By identifying mentions of symptoms, behaviors, and health conditions, NLP allows researchers to extract meaningful information from noisy, longitudinal text streams. This is particularly valuable when studying platforms like Facebook, where individual users may have years of diverse content, only some of which is relevant to health. In such settings, NLP-based classification models can be used to automatically identify and flag posts related to a given condition, facilitating large-scale, efficient analysis of patient-generated narratives.

While prior work has applied these tools to broad medical topics, the extent to which social media and NLP can jointly illuminate specific neurological conditions, including Parkinson’s disease (PD), remains underexplored. PD, the second-most common neurodegenerative disorder, affects over 6.2 million people worldwide [9]. Its clinical heterogeneity, spanning both motor and non-motor symptoms, complicates diagnosis and progression modeling [10]. PD is diagnosed based on motor symptoms that emerge after extensive dopaminergic neurodegeneration, often estimated to exceed 50% neuronal loss [11]. The prodromal phase, during which neurodegeneration occurs before clinical diagnosis, remains difficult to study prospectively, as most PD cases are idiopathic [12]. Leveraging retrospective data sources that document lived experiences before and after diagnosis may provide insights into the premorbid period and early disease manifestations.

Social media-based research in PD has taken several directions including identifying potential drug repurposing candidates through patient-reported adverse effects [13], evaluating the quality of PD-related health information available to patients on platforms like YouTube [14], and quantifying the scale and structure of PD-related online communities on Facebook and Twitter [15]. Other studies have analyzed passive behavioral data including time spent on social media to track indicators such as social withdrawal and quality of life [16] and examined the sentiment of PD-related tweets [17]. Some researchers have sought to understand the content and nature of patient discourse. For instance, Chu and Jang [18] analyzed posts from a large Korean online community to identify unmet information needs, with a focus on medications, non-motor symptoms, and treatment decisions. They found that caregivers seek and share a lot of information about their loved ones with PD. Damier et al. [8] conducted a multi-country study of public social media posts, revealing recurring themes around disease burden, symptom fluctuations, and caregiver stress. Little et al. [19] demonstrated the potential of structured self-reported data from PatientsLikeMe to capture high-frequency symptom fluctuations not typically detected in clinical trials.

While these studies demonstrate the value of social media and user-generated content in PD research, they are typically limited by reliance on either scraped public data or structured, self-selected registries. In most cases, researchers do not know the identity of individual users, cannot follow them longitudinally, or lack access to the full trajectory of their social media activity [8,13,14,15,17,18,19].

This study introduces a novel approach: we analyze full historical Facebook data donated by a clinically characterized cohort of individuals with PD, essential tremor (ET), atypical parkinsonism (AP), and their caregivers. This design enables us to examine real-world, unsolicited health disclosures shared both before and after diagnosis—a temporal window rarely accessible in neurological research. In contrast to previous studies, we can link user-level data across time, identify PD-related posts at scale using NLP, and assess whether longitudinal Facebook activity reflects disease emergence or progression. This retrospective view of premorbid behavior in a known cohort lays the groundwork for future efforts to use digital traces in early disease detection.

By demonstrating the feasibility and value of using real-world, longitudinal Facebook data in PD research, this work expands the scope of what social media platforms can reveal about disease-related experiences and disclosures.

## 2. Materials and Methods

### 2.1. Participants and Data Collection

Participants were recruited through the Emory Brain Health Center, research recontact lists, and PD-specific community events. Eligible participants included individuals diagnosed with PD, ET, or AP, as well as caregivers of individuals with those conditions. We included individuals with ET and AP because PD shares clinical features with and is sometimes misdiagnosed as these conditions [20]. Given caregivers are deeply involved in the emotional and logistical aspects of PD management, they were included to capture the broader landscape of PD-related discourse on social media. Participants had to communicate in English. No additional exclusion criteria were applied. Written informed consent was obtained in person or via teleconferencing, following protocols approved by Emory University’s Institutional Review Board (STUDY00005722) and the Declaration of Helsinki.

During structured interviews, demographic and clinical information was collected. Disease severity and symptom burden in PD and AP participants were assessed using the Movement Disorder Society–Unified Parkinson’s Disease Rating Scale (MDS-UPDRS) Parts I (non-motor symptoms), II (motor symptoms), and IV (motor complications) [21], where higher scores indicate greater impairment. ET participants were assessed using MDS-UPDRS Parts I and II, as motor complications (Part IV) are not relevant to ET. MDS-UPDRS Part III (motor examination) was not collected, as physical examinations were outside the scope of data collection.

Participants who opted to share their Facebook data were guided through downloading their Posts, Comments and Reactions, Pages, and Groups in JSON format. They were instructed to download their complete account history, capturing data from account creation through study participation. All text-based content—posts, comments, media captions, and other written engagement—was authored by the account owner and collectively referred to as “posts”. Private messages were neither collected nor analyzed. Study staff provided technical assistance as needed. All data were securely uploaded to a HIPAA-compliant REDCap database [22].

### 2.2. PD-Related Post Identification

#### 2.2.1. Text Extraction from JSON Files

Participant-generated text was extracted from Facebook data exports. Given that individual JSON files were not mutually exclusive, deduplication was necessary. Exact duplicates were removed based on identical timestamps and text, and near-duplicates were filtered by retaining only the earliest instance unless at least 180 s had elapsed between occurrences, ensuring that only intentionally reshared content was preserved.

Posts flagged as shared memories were retained, as they represented intentional reposts, but associated in-text timestamps (e.g., “X years ago”) were removed to prevent redundancy. Text encoding was standardized, and the final dataset was stored in a HIPAA-compliant REDCap database [22].

#### 2.2.2. PD-Related Term Dictionary and Search Strategy

A PD-specific term dictionary with over 1000 terms was developed based on the supplementary appendix from Bloem et al. [10], a comprehensive clinical review of Parkinson’s disease published in The Lancet, which outlines motor and non-motor symptoms across all stages of PD. To extend coverage beyond formal medical terminology, we supplemented this list with patient-facing terms from WebMD’s Parkinson’s disease drug treatment page [23], which provided commonly prescribed medications and associated lay descriptions. We also incorporated terminology from our prior work on fall-related disclosures in PD [24], in which individuals described their fall experiences in their own words. These narratives revealed real-world vocabulary and contextual language (e.g., specific locations, causes, and emotional framing of falls). Including this language ensured better sensitivity to fall-related content as it might naturally appear on Facebook. Finally, we used ChatGPT-4 to generate additional colloquial variants and manually reviewed the combined list to optimize coverage of both clinical and informal expressions. See Appendix A for the full dictionary.

This term dictionary was used to identify posts that may be related to PD. To enable consistent matching and reduce linguistic variability, we preprocessed the text by converting all characters to lowercase, removing punctuation and common filler words (referred to as “stopwords”, e.g., “the”, “and”), and reducing words to their root forms using stemming (e.g., “trembling” becomes “trembl”) [25]. Posts were then flagged if they contained any match to either the stemmed or unstemmed version of a dictionary term.

#### 2.2.3. Development of Ground-Truth Dataset

To train a supervised machine learning model, we required a ground-truth dataset in which human reviewers labeled whether each post was relevant to PD. Keyword matching alone can be noisy and overly inclusive—for example, both “I fell down” and “I fell in love” would be flagged, although only the former may indicate a PD-related fall event. To address this, we manually labeled a subset of posts that contained at least one term from the PD-specific dictionary.

This subset consisted of all flagged posts from an initial group of enrolled participants, selected pragmatically as data became available. The selection process was not randomized or stratified across participants.

Posts were included if they explicitly mentioned PD, related disorders, treatments, symptoms, advocacy events, or lacked sufficient context to rule out a PD connection (e.g., “I had a follow-up with my doctor today”). Posts were excluded only if sufficient context indicated they were unrelated (e.g., “I am so fatigued from COVID”). Given that both caregivers and individuals with PD may discuss others with PD, posts were evaluated for general relevance to PD, not just the poster’s personal experience.

Each post was independently assessed by two trained reviewers for relevance to PD using a high-sensitivity approach. One reviewer labeled all posts, while the second review was divided between two additional trained annotators. Inter-rater reliability was evaluated using Cohen’s Kappa [26]. Discrepancies were resolved through consensus discussion to ensure consistent and accurate labeling.

#### 2.2.4. Classifier Development

The dataset was randomly split into 80% training and 20% testing. All feature transformations, including vectorization, scaling, and encoding, were derived from the training set and applied to the test set. Posts were cleaned by removing URLs, tags, hashtags, special characters, punctuation, extra spaces, and stopwords. The processed text was then represented in two ways. First, lemmatization was performed using the NLTK WordNet lemmatizer [27] without explicit part-of-speech tagging, meaning words were lemmatized assuming noun forms by default. Lemmatized text—where words are reduced to their base or dictionary form (e.g., “trembling” becomes “tremble”)—was transformed into numeric features using a technique called Term Frequency–Inverse Document Frequency (TF-IDF). This approach assigns higher weight to words that are common in a given post but rare across the dataset, highlighting distinctive language. We included single words, as well as word pairs (bigrams) and triplets (trigrams), to capture short phrases (e.g., “muscle pain” or “falling down”). Terms that appeared in fewer than two posts were excluded to reduce noise from rare or potentially irrelevant language. Second, tokens were mapped to cluster-based embeddings using a word-clustering approach derived from a Twitter-based corpus [28]. Clusters were vectorized using TF-IDF, capturing only single clusters and applying the same frequency threshold.

Model features included vectorized lemma and cluster representations, normalized age at time of posting, and one-hot encoded categorical variables (gender, PD diagnosis status). Multiple classifiers, including K-Nearest Neighbors (KNNs), Support Vector Machines (SVMs), Random Forest, AdaBoost, Naïve Bayes, Decision Trees, and XGBoost, were trained using five-fold cross-validation. To address class imbalance, we applied stratified cross-validation using StratifiedKFold, which ensures each fold preserves the original class distribution. GridSearchCV [29] was used to optimize macro-averaged recall, ensuring balanced sensitivity across PD-related and non-PD content. All models used the default classification threshold of 0.5; no post hoc thresholding was applied. We also experimented with a soft-voting ensemble that combined all classifiers above.

Performance was evaluated using macro-averaged recall, precision, and F_1_-score to account for class imbalance. 95% confidence intervals (CIs) were estimated via bootstrapping, and model discrimination was assessed using Receiver Operating Characteristic (ROC) curves and Area Under the Curve (AUC) scores. The classifier with the highest recall was applied to the full dataset.

### 2.3. Analyses

Descriptive statistics were used to summarize participant demographics and clinical characteristics. Continuous variables are reported as the mean ± standard deviation (SD), and categorical variables as counts and percentages. Group comparisons between Facebook users and non-users were conducted using Welch’s *t*-tests for continuous variables and Fisher’s exact tests for categorical variables.

Facebook engagement was quantified by calculating each participant’s total number of posts and duration of account activity (i.e., time between earliest and most recent post). For participants with movement disorders, diagnosis dates were collected directly. Although diagnosis dates were not formally collected from caregivers, many participated alongside the individuals they cared for, enabling inferred diagnosis timing and approximate pre/post comparisons in those cases.

To account for individual differences in Facebook activity, we normalized the quantity of PD-related content by post volume rather than account age. This approach provides a more accurate measure of engagement, as some participants had long-standing accounts but posted infrequently. Specifically, we computed the percentage of PD-related posts as follows:

Percentage = (Number of PD-related posts)/(Total number of posts) × 100

These percentages were calculated for three timeframes: overall, pre-diagnosis, and post-diagnosis.

PD-related posts were identified using a recall-optimized Naïve Bayes classifier, applied to posts containing at least one term from our term dictionary. Additional rule-based methods flagged explicit mentions of “Parkinson” or the standalone abbreviation “PD” using the regular expression \bPD\b to prevent false matches (e.g., “updated”).

Wilcoxon signed-rank tests were conducted to evaluate within-subject changes in the percentage of PD-related posts before and after diagnosis. Analyses were performed separately for individuals with PD and for caregivers and were limited to participants with valid, non-identical pre/post percentages. Parallel analyses were conducted using a filtered post set that excluded exercise-related content, to assess whether physical activity references disproportionately influenced results.

To assess whether individuals with PD posted more PD-related content prior to diagnosis than a comparison group, we evaluated differences in pre-diagnosis percentages between participants with PD and caregivers. Participants with no Facebook activity prior to diagnosis were excluded from this analysis. Normality was assessed using the Shapiro–Wilk test, and due to non-normal distribution in the PD group, the Mann–Whitney U test was used for between-group comparisons.

## 3. Results

### 3.1. Dataset Characteristics and Participant Engagement

#### 3.1.1. Recruitment and Demographics

We attempted to contact 369 individuals and successfully reached 139. During phone screenings, participants were asked whether they had a Facebook account. Of those contacted, 59% reported having a Facebook account, while 20% did not specify their Facebook status. Among individuals who confirmed having a Facebook account, 85% agreed to participate.

Of the 139 individuals reached, 62 enrolled and 60 completed the interview. The final dataset included 38 individuals with PD, 4 with ET, 3 with AP, and 15 caregivers. The sample was composed of 43% women, with an average age of 66.53 ± 12.99 years. Most participants were white (80%), non-Hispanic or Latino (93%), and college-educated (78%). Among participants with PD (N = 38), the average age was 69.21 ± 10.11 years. Disease characteristics included an average disease duration of 8.62 ± 4.73 years, an MDS-UPDRS Part I score of 14.03 ± 7.48, an MDS-UPDRS Part II score of 13.81 ± 9.68, and an MDS-UPDRS Part IV score of 6.25 ± 4. See Table 1 for a full breakdown of participant demographics stratified by disease status.

Among the 60 participants, 46 shared their Facebook data. Eleven participants did not have Facebook accounts and three had Facebook accounts but did not complete the data-sharing process. Of those who shared Facebook data, 30 had PD, 3 had ET, 1 had AP, and 12 were caregivers.

#### 3.1.2. Comparison of Facebook Users and Non-Users

We compared Facebook users and non-users based on demographics and clinical status. Participants with Facebook accounts were significantly younger than those without accounts (64.46 ± 13.24 vs. 75.72 ± 6.41 years; t(32.14) = 4.16, *p* < 0.001, d = −0.91). However, there were no significant differences between Facebook users and non-users in gender (*p* = 0.09), race (*p* = 0.49), ethnicity (*p* = 1.00), or education level (*p* = 0.34).

Among participants with PD (N = 30), those with Facebook accounts were also significantly younger than those without (67.24 ± 9.89 vs. 77.96 ± 5.69 years; t(15.52) = 3.84, *p* = 0.002, d = −0.46). Facebook users and non-users with PD did not differ in MDS-UPDRS Part I score (*p* = 0.86), Part II score (*p* = 0.32), Part IV score (*p* = 0.39), or disease duration (*p* = 0.81).

#### 3.1.3. Facebook Account History

Among the 46 participants who shared Facebook data, one had an account but never generated or shared any content, resulting in empty JSON files. This participant was included in overall Facebook user counts but excluded from subsequent analyses. All reported results are based on the remaining 45 participants.

**Figure 1:**
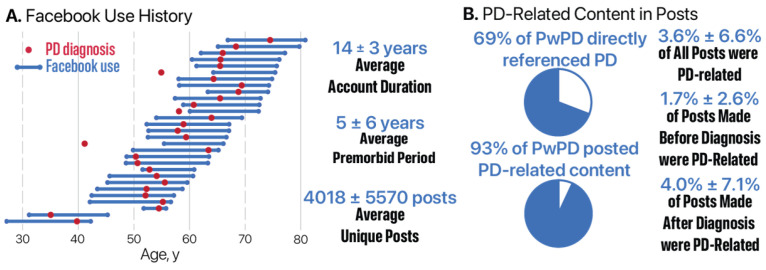
Facebook use and PD-related content among participants with Parkinson’s disease (PwPD). (**A**) Facebook timelines for PwPD (N = 29), with blue lines showing posting history and red dots marking age at diagnosis. (**B**) Proportion of PD-related posts overall, pre-diagnosis (N = 26), and post-diagnosis (N = 29), including rates of explicit PD mention and classifier-flagged content.

On average, participants had used Facebook for 13 ± 3 years and posted 4491 ± 5637 times. Among those with PD (N = 29), the average length of Facebook use was 14 ± 3 years, with an average of 4018 ± 5570 posts. On average, participants with PD had used Facebook for 5 ± 6 years before their diagnosis, with 90% creating their account before diagnosis (see Figure 1A).

### 3.2. Identification of PD-Related Posts

#### 3.2.1. Ground-Truth Dataset

The ground-truth dataset consisted of 6750 posts written by 14 individuals with PD and 5 caregivers. Annotators achieved substantial interrater reliability (Cohen’s Kappa = 0.79) [26] when labeling the posts. Among the 6750 reviewed posts, 2400 (35.6%) were classified as PD-related, while 4350 (64.4%) were deemed irrelevant. This imbalance was partly due to keyword ambiguity, where certain terms appeared in non-PD contexts.

#### 3.2.2. Classifier Performance

Table 2 presents the macro-averaged classification metrics for each model. The Naïve Bayes classifier achieved the highest recall (0.86 95% CI: [0.84–0.88]). Although the soft-voting ensemble was implemented, the Naïve Bayes classifier was selected for final PD-related post identification due to its superior recall, ensuring sensitivity to PD disclosures. Please see Appendix B for model hyperparameters.

**Figure 2:**
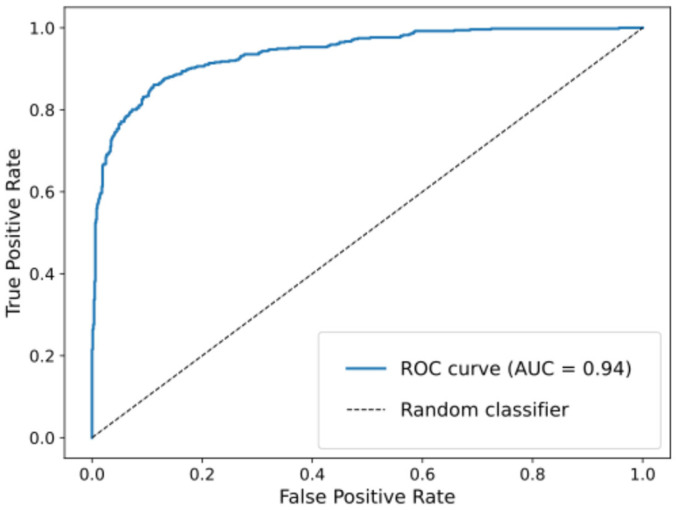
ROC curve of Naïve Bayes classifier to identify PD-related posts.

Overall model discrimination was strong, with an AUC of 0.94 (Figure 2), indicating high separability between PD-related and irrelevant posts.

### 3.3. PD-Related Facebook Activity

#### 3.3.1. Prevalence of PD-Related Posts by Diagnosis Group and Timeframe

Using the Naïve Bayes classifier, we identified posts containing PD-related content across participant Facebook timelines. Overall, 96% of participants had at least one post flagged as PD-related and 67% of all participants explicitly referenced PD at least once. Among individuals with PD, 69% explicitly mentioned PD at least once, and 93% had at least one post flagged as PD-related (see Figure 1B).

Table 3 summarizes the percentage of PD-related posts relative to each participant’s total Facebook activity, stratified by diagnosis group and diagnosis phase. Individuals with PD had an average of 3.6% ± 6.6% of posts classified as PD-related, increasing from 1.7% ± 2.6% pre-diagnosis to 4.0% ± 7.1% post-diagnosis. Among caregivers, the overall PD-related percentage was slightly lower (2.6% ± 5.6%), with minimal change between the pre-diagnosis (1.1% ± 1.1%) and post-diagnosis (1.0% ± 1.1%) periods. Note that pre- and post-diagnosis values are based on a subset of caregivers for whom diagnosis date information was available, resulting in smaller sample sizes than those reported for overall activity. Table 3 also summarizes the percentage of PD-related activity, excluding posts related to exercise.

#### 3.3.2. Within-Group Changes in PD-Related Posting After Diagnosis

We next evaluated whether PD-related posting increased following diagnosis. Among individuals with PD, a Wilcoxon signed-rank test showed a statistically significant increase in PD-related content after diagnosis (N = 27; W = 105.0, *p* = 0.044). This trend remained marginally significant when exercise posts were excluded (N = 24; W = 83.0, *p* = 0.056), suggesting that increased PD engagement was not solely attributable to fitness discussions.

In contrast, caregivers did not show significant within-subject changes in PD-related posting across the diagnostic boundary, regardless of whether exercise content was included (N = 9; W = 16.0, *p* = 0.496) or excluded (N = 6; W = 7.0, *p* = 0.563).

Due to the small sample sizes of the ET and AP groups, we did not conduct statistical analyses for these participants. However, we note that the individual with AP showed an increase in PD-related posts (excluding exercise) from 0.3% pre-diagnosis to 3.8% post-diagnosis. This pattern aligns with his clinical diagnosis of post-traumatic parkinsonism.

#### 3.3.3. Between-Group Comparisons Before and After Diagnosis

We tested for age matching to evaluate the suitability of caregivers as a comparison group. A Welch’s two-sample *t*-test indicated no significant age difference between PD participants and caregivers who shared Facebook data (t(14.76) = −1.24, *p* = 0.23, 95% CI [−15.63, 4.13]).

We compared PD-related posting rates between participants with PD and caregivers across diagnosis phases. To assess pre-diagnosis differences, we restricted analysis to participants with Facebook activity prior to diagnosis (N = 26 PwPD, N = 7 caregivers). A Mann–Whitney U test revealed no statistically significant difference between groups (U = 95.00, *p* = 0.88). There was also no significant difference between participants with PD and caregivers in the percentage of PD-related posts made after diagnosis (U = 170.00, *p* = 0.18).

#### 3.3.4. Thematic Shifts in PD Discourse over Time

**Figure 3:**
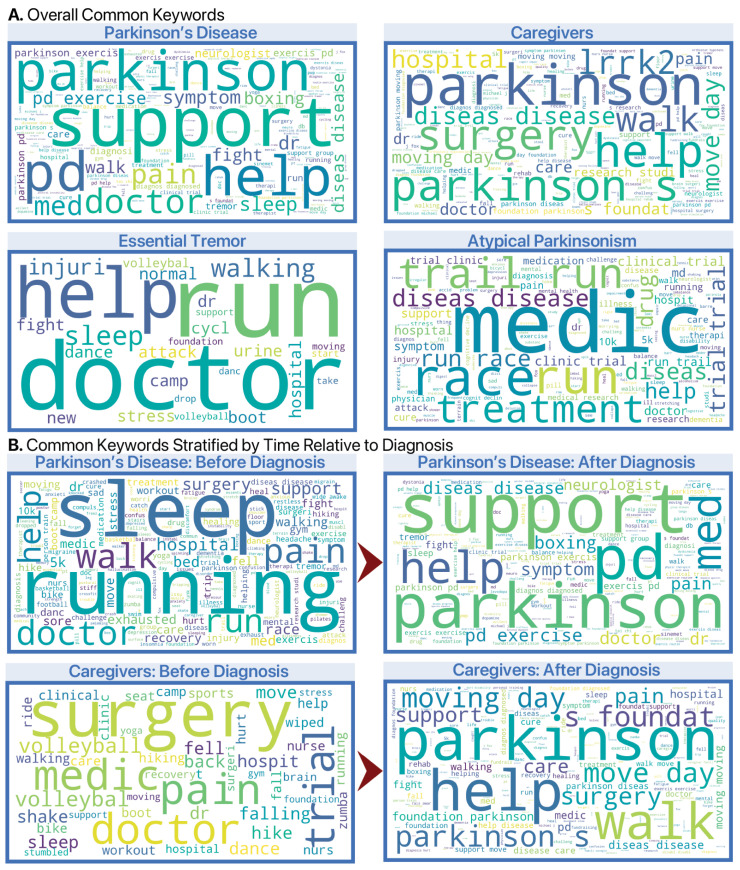
Word clouds of PD-related keywords in Facebook posts. Word clouds show the most frequently used keywords in PD-related Facebook posts, grouped by diagnosis group and diagnosis phase. Larger words indicate higher frequency. (**A**) Overall word clouds for Parkinson’s disease, caregivers, atypical parkinsonism, and essential tremor. (**B**) Phase-specific word clouds (pre- and post-diagnosis) for Parkinson’s disease and caregiver groups show how content changes following diagnosis.

To qualitatively explore content patterns, we generated word clouds of the most frequent keywords in PD-related posts (Figure 3). These visualizations illustrate distinct differences by diagnosis group and phase.

Among individuals with PD, post-diagnosis content emphasized clinical and management topics such as PD, exercise, support, and neurologist. Pre-diagnosis content more frequently referenced general health and symptom terms such as pain, sleep, and hospital, potentially reflecting early prodromal experiences.

In contrast, caregivers’ pre-diagnosis posts included few PD-related terms. Post-diagnosis, their posts increasingly featured caregiving and advocacy-related terms like parkinson, moving day, support, and foundation, reflecting a shift in online behavior once PD became salient in their lives.

Word clouds for participants with ET and AP showed limited overlap with PD-specific vocabulary, although some references to health and mobility were observed.

## 4. Discussion

### 4.1. Principal Findings

#### 4.1.1. Recruitment Feasibility and Platform Representation

This study demonstrates the feasibility of using Facebook as a data source for PD research. Among successfully contacted individuals with Facebook accounts, 85% agreed to share their data, confirming that many participants are willing to contribute comprehensive social media histories for scientific purposes. This willingness highlights the potential of participant-donated social media to support research into chronic disease experiences.

However, the generalizability of social media–based research remains limited. Digital literacy and internet access vary widely by demographic [30], and our sample was predominantly White and college-educated. Facebook users in the study were significantly younger than non-users, which may reflect broader generational differences in social media engagement.

Although PD severity and disease duration did not significantly differ between Facebook users and non-users, this may reflect selection bias. Individuals with milder disease may be more likely to participate in research requiring digital engagement; all but one study sessions occurred over Zoom. Moreover, Facebook’s data-sharing protocol requires users to download and submit their own archives. While this process enhances ethical transparency by ensuring participants retain control over their data, it introduces a technical burden that may deter individuals with limited digital literacy or more advanced disease. In our study, some participants were unable to complete the consent process, and others enrolled but ultimately did not submit data, citing the process as too cumbersome. This highlights a tradeoff between ethical integrity and accessibility that future research must address.

The perspectives captured in this study likely reflect individuals who are more digitally literate, motivated to engage in research, and capable of navigating a multistep data-sharing process. These characteristics may shape both the quantity and type of PD-related content available for analysis. Still, even within this relatively tech-savvy group, content-sharing behaviors varied widely. Some individuals contributed hundreds of PD-related posts, while 4% of participants overall—and 7% of those with PD—did not share any posts flagged as PD-related. This variability suggests that privacy preferences and disclosure norms differ even among participants with similar levels of digital access, adding nuance to how social media data reflect lived experience.

#### 4.1.2. Longitudinal Engagement and Health-Related Disclosures

This study demonstrates that individuals with PD maintain long-term and active engagement with Facebook, offering a rich timeline of everyday experiences and health-related disclosures. On average, participants with PD had Facebook accounts spanning 14 years and authored over 4000 unique posts. Notably, 90% had joined the platform before their diagnosis, providing a valuable window into pre- and post-diagnostic life.

While PD-related content represented a small fraction of overall activity (3.6% on average), its presence across such a large volume of posts highlights the potential for social media to capture meaningful aspects of the PD experience. Nearly all participants with PD (93%) had at least one post flagged as PD-related by our classifier, and 69% explicitly mentioned PD by name. This finding underscores that individuals are willing to disclose health information online, even in general-purpose platforms like Facebook that are not tailored for health communication, and is consistent with the prior literature [8,18,19].

We also observed a significant increase in the proportion of PD-related posts following diagnosis, supporting the idea that health disclosures intensify once a formal diagnosis is received. This increase persisted even when exercise-related content was excluded, although it dropped to a trending level of significance. This trend suggests that while broader PD engagement rises post-diagnosis, exercise plays a notable role in driving this increase. Given that physical activity is a central component of PD management and contributes meaningfully to quality of life [10], it is not surprising that exercise-related content would feature prominently in post-diagnostic social media activity. These findings highlight how lifestyle-based interventions—like exercise—not only shape clinical care but also influence how people choose to represent and cope with illness in digital spaces.

While this study focused on individuals with PD, patterns among caregivers and participants with ET or AP offer important context. Caregivers posted less PD-related content than individuals with PD and showed no significant increase in posting after diagnosis. This suggests that PD-related social media engagement among caregivers may emerge reactively, often after diagnosis is established, and reflects advocacy or support roles rather than personal health disclosure. The limited availability of diagnosis dates also constrained caregiver-specific temporal analyses.

ET participants showed minimal PD-related content and no notable changes over time, as expected for individuals without PD. These patterns support their use as a contrast group, although the sample size was too small for statistical comparison.

The single AP participant showed an increase in PD-related posts from 0.3% to 3.8% (excluding exercise) following diagnosis, resembling the trend observed in the PD group. This aligns with his clinical history of post-traumatic parkinsonism, where the onset of parkinsonian symptoms is abrupt as a result of trauma [31].

Although limited by small sample sizes, these findings support meaningful differences in how PD-related content emerges across groups and highlight areas for future investigation.

#### 4.1.3. Early Signals and the Limits of Specificity

Although some individuals with PD posted about health concerns before diagnosis, the proportion of PD-related content in the pre-diagnosis period did not significantly differ from that of caregivers. Both groups showed low—but nonzero—levels of PD-related posts prior to diagnosis. This finding suggests that while prodromal symptoms may surface on social media, similar language also appears in non-patient contexts, limiting the specificity of such signals for early detection.

Qualitatively, individuals with PD more frequently used symptom-related terms before diagnosis—such as “sleep”, “pain”, and “tremor”—while caregivers tended to reference general health or fitness topics, such as “volleyball”. These differences hint at potential early indicators of disease, but a more comprehensive thematic analysis is needed to determine whether prodromal language patterns can be reliably distinguished from typical social media discourse. Large language models may be particularly adept at this research task.

We also explored whether excluding exercise-related content altered the presence of early PD signals. Although exercise was a prominent theme—especially after diagnosis—its removal did not eliminate PD-related language in the pre-diagnosis period. This suggests that early PD-related discourse involves more than just fitness-related posts and may include symptoms or medical experiences.

Finally, it is important to note that our study lacked a truly unaffected, age-matched control group. While caregivers are often used as comparators, their proximity to the person with PD—even before diagnosis—may influence their social media behavior. We caution against treating them as naïve controls. Future work should incorporate healthy participants with no personal or familial connection to PD to better evaluate whether early online engagement patterns can meaningfully indicate disease onset.

#### 4.1.4. Content Evolution and Behavioral Patterns

The temporal evolution of PD-related discourse illustrates how health conditions become increasingly salient in a person’s social media behavior. Prior to diagnosis, individuals with PD posted more general symptom-related or lifestyle content (e.g., pain, sleep, hospital visits), while post-diagnosis posts became more focused on disease management, treatment, and support systems. These behavioral shifts provide a digital trace of the illness experience that complements clinical documentation.

Importantly, PD-related content was not limited to those with the diagnosis. The presence of such content in caregiver posts, especially post-diagnosis, reinforces the idea that social media reflects shared illness experiences. This finding is consistent with previous work outlining the important role caregivers play on forums related to PD care [18]. These behavioral patterns underscore the potential of social media data not just for early detection, but for understanding how individuals and their communities adapt over time.

#### 4.1.5. Methodological Considerations and Future Directions

Our approach combined a PD-specific dictionary with a Naïve Bayes classifier optimized for recall to identify PD-related posts. While effective for detecting explicit mentions, this method likely misrepresents the full scope of PD-related discourse due to its reliance on surface features.

Our reliance on keyword-matching introduced limitations in the precision of post classification. Posts lacking explicit terminology may have been missed, leading to underestimation of relevant discourse. At the same time, our labeling approach prioritized sensitivity over specificity—posts were included if they explicitly referenced PD or if there was insufficient context to rule out a PD connection (e.g., “I went to the doctor today”). This conservative strategy may have led to the overinclusion of posts not truly reflective of PD-related concerns, particularly in the pre-diagnosis period, where nonspecific health-related content could be misinterpreted as early PD signals. As such, both false negatives and false positives are possible.

Future work should explore more nuanced, context-aware models—such as large language models or deep learning approaches—to better capture implicit references and emotional tone. For example, LLMs could help disambiguate posts where general health terms are used metaphorically or non-medically (e.g., ‘I’m so tired’ vs. fatigue as a symptom), and could enable temporal modeling of symptom trajectories by extracting structured symptom mentions across timepoints.

Overall, while the potential of social media to support early detection remains promising, our findings highlight the importance of specificity, appropriate comparison groups, and contextual interpretation. Facebook data offer a valuable lens into longitudinal health behavior, disease salience, and the broader social narrative of illness. As research in this area advances, it will be critical to develop rigorous, representative designs that move beyond feasibility toward clinical and public health relevance. In the future, social media–derived insights could complement clinical tools by contributing to digital phenotyping efforts or integrating with early risk stratification models. For example, longitudinal trends in symptom mentions or shifts in social engagement could be passively monitored alongside clinical assessments to support early detection or enhance ongoing disease monitoring, especially when traditional data sources are limited or inaccessible.

### 4.2. Ethical Considerations

This study highlights the ethical advantages of our Facebook-based approach, where participants actively consented to data sharing rather than having their posts passively scraped via an Application Programming Interface (API)—a tool that allows external programs to automatically access and retrieve user data from a platform, often without the user’s direct involvement. Social media research on platforms like Reddit typically involves extracting user-generated content without direct user awareness, even if permitted by the platform’s terms and conditions. Following the Cambridge Analytica scandal, Facebook restricted API access [32], leading us to collect data directly from account owners instead. While this method enhances ethical integrity, it limits scalability and restricts datasets to unidirectional conversations, as only account-owner content is retrievable. Future work should explore ways to balance participant agency with efficient data collection.

## 5. Conclusions

This study establishes Facebook as a feasible and ethically sound data source for PD research, demonstrating that individuals share PD-related information both before and after diagnosis. These findings highlight social media’s potential for disease monitoring and early detection. Further refinement of computational methods, an inclusion of a naïve control group, and integration with clinical data could enhance the utility of social media-derived insights.

## Figures and Tables

**Table 1:** Participant demographics.

Variable	Parkinson’s Disease (N = 38)	Essential Tremor (N = 4)	Atypical Parkinsonism (N = 3)	Caregivers (N = 15)	Total (N = 60)
Age in years, mean (SD)	69.2 (10.1)	54.1 (25.1)	68.6 (15.8)	62.6 (13.8)	66.5 (13.0)
Women, N (%)	13 (34%)	0 (0%)	1 (33%)	12 (80%)	26 (43%)
Race N (%)					
African American/Black	6 (16%)	0 (0%)	1 (33%)	1 (7%)	8 (13%)
Asian	0 (0%)	0 (0%)	0 (0%)	2 (13%)	2 (3%)
White	32 (84%)	4 (100%)	1 (33%)	11 (73%)	48 (80%)
More Than One Race	0 (0%)	0 (0%)	1 (33%)	1 (7%)	2 (3%)
Ethnicity					
Hispanic or Latino	0 (0%)	0 (0%)	1 (33%)	0 (0%)	1 (2%)
Not Hispanic or Latino	35 (92%)	4 (100%)	2 (67%)	15 (100%)	56 (93%)
Unknown/Not Reported	3 (8%)	0 (0%)	0 (0%)	0 (0%)	3 (5%)
Completed Education					
High School	3 (8%)	1 (25%)	1 (33%)	1 (7%)	6 (10%)
Junior College	5 (13%)	0 (0%)	0 (0%)	2 (13%)	7 (12%)
College	16 (42%)	3 (75%)	0 (0%)	5 (33%)	24 (40%)
Graduate Degree	14 (37%)	0 (0%)	2 (67%)	7 (47%)	23 (38%)
Shared Facebook data	30 (79%)	3 (75%)	1 (33%)	12 (80%)	46 (77%)
Disease Duration in years, mean (SD)	8.6 (4.7)	14.7 (6.8)	5.2 (0.7)	-	-
MDS UPDRS Part 1					
Mean (SD)	14.0 (7.5)	4.5 (3.1)	10.0 (9.6)	-	-
N-Miss	2	0	0	-	-
MDS UPDRS Part 2					
Mean (SD)	13.8 (9.7)	2.5 (3.0)	18.3 (17.0)	-	-
N-Miss	2	0	0	-	-
MDS UPDRS Part 4					
Mean (SD)	6.2 (4.0)	-	5.3 (6.8)	-	-
N-Miss	2	-	0	-	-

**Table 2:** Classifier performance: macro-averaged metrics.

Model	Recall (95% CI)	Precision (95% CI)	F1-Score (95% CI)
Naïve Bayes	**0.86 (0.84–0.88)**	0.89 (0.87–0.90)	**0.87 (0.85–0.89)**
Ensemble	0.84 (0.82–0.86)	**0.90 (0.88–0.91)**	0.86 (0.84–0.88)
SVM	0.84 (0.82–0.86)	0.86 (0.84–0.88)	0.85 (0.83–0.87)
Decision Tree	0.81 (0.78–0.83)	0.83 (0.81–0.85)	0.81 (0.79–0.84)
Random Forest	0.79 (0.77–0.81)	0.87 (0.85–0.89)	0.81 (0.78–0.83)
XGBoost	0.79 (0.77–0.81)	0.87 (0.85–0.89)	0.81 (0.78–0.83)
KNN	0.77 (0.75–0.79)	0.83 (0.81–0.85)	0.79 (0.76–0.81)
AdaBoost	0.72 (0.70–0.74)	0.85 (0.83–0.87)	0.73 (0.70–0.75)

Values represent macro-averaged performance metrics with 95% confidence intervals. Bolded values indicate the highest score achieved for each metric across all models.

**Table 3:** Percent of PD-related posts on Facebook relative to overall activity.

	Overall		Before Diagnosis		After Diagnosis	
Group	N	Percent ± STD	N	Percent ± STD	N	Percent ± STD
PD	29	3.6% ± 6.6%	26	1.7% ± 2.6%	29	4.0% ± 7.1%
ET	3	0.8% ± 0.1%	3	1.0% ± 0.1%	3	0.7% ± 0.1%
AP	1	5.1% ± NA	1	9.3% ± NA	1	4.9% ± NA
CG	12	2.6% ± 5.6%	7	1.1% ± 1.1%	9	1.0% ± 1.1%
Excluding Exercise-Related Posts						
PD	29	2.2% ± 3.6%	26	1.2% ± 2.5%	29	2.2% ± 4.0%
ET	3	0.5% ± 0.1%	3	0.2% ± 0.3%	3	0.5% ± 0.0%
AP	1	3.6% ± NA	1	0.3% ± NA	1	3.8% ± NA
CG	12	1.7% ± 4.0%	7	0.8% ± 1.0%	9	0.4% ± 0.6%

Abbreviations: PD = Parkinson’s Disease; ET = Essential Tremor; AP = Atypical Parkinsonism; CG = Caregiver. Note that pre- and post-diagnosis Ns may differ due to missing or post-onset data.

## Data Availability

Code supporting the project, including text extraction from Facebook exports, keyword flagging, classifier development, application, and analyses, is available at https://github.com/jeannempowell/JCM_pd-on-facebook. However, the dataset and trained models cannot be shared due to the risk of retaining identifiable information, making full de-identification infeasible. During the preparation of this manuscript/study, the authors used ChatGPT4o for the purposes of copyediting. The authors have reviewed and edited the output and take full responsibility for the content of this publication.

## Abbreviations

The following abbreviations are used in this manuscript:

API	Application Programming Interface
AP	Atypical Parkinsonism
AUC	Area Under the Curve
CI	Confidence Interval
ET	Essential Tremor
HIPAA	Health Insurance Portability and Accountability Act
JSON	JavaScript Object Notation
KNN	K-Nearest Neighbor
MDS-UPDRS	Movement Disorder Society-Unified Parkinson’s Disease Rating Scale
PD	Parkinson’s disease
PwPD	People with Parkinson’s disease
ROC	Receiver Operating Characteristic
SVM	Support Vector Machine
TF-IDF	Term Frequency–Inverse Document Frequency

## Appendix A

{“constipation”: [“poor bowel habits”, “digestive blockage”, “need more fiber”, “bowel issues”, “blocked up”, “stool inconsistency”, “constipation”, “stomach cramps”, “bowel discomfort”, “irregularity”, “intestinal sluggishness”, “laxative”, “cramping”, “irregular bowel movements”, “digestive upset”, “digestive issues”, “digestive slowdown”, “gut health concerns”, “gastrointestinal issues”, “bowel trouble”, “bowel difficulty”, “stool”, “stomach pain”, “digestive problems”, “abdominal bloating”, “infrequent defecation”, “straining to defecate”, “bowel obstruction”, “irregular bowels”, “bowel”, “poop”, “colon concerns”, “infrequent bowel movements”, “constipated”, “bowel movement trouble”, “hard stools”, “slow bowel transit”, “stool softener”, “laxative use”, “infrequent stools”, “intestinal discomfort”, “difficulty passing stool”, “digestive distress”, “lack of fiber”, “stool problems”, “slow digestion”, “stool issues”, “fecal impaction”, “struggle in the bathroom”],

“REM sleep behavior disorder”: [“violent dreaming”, “bed partner disturbance”, “hitting during sleep”, “restless nighttime behavior”, “sleep aggression”, “nighttime agitation”, “sleep disruptions”, “violent dreams”, “dream reenactment”, “unusual sleep actions”, “vocalization in sleep”, “sleep behavior disorder”, “punching in sleep”, “nighttime episodes”, “sleep walking”, “physical dream expression”, “REM sleep behavior disorder”, “dream enactment”, “sleepwalking”, “sleep movement”, “sleep disturbance”, “physical activity during sleep”, “acting out while asleep”, “violent sleep behavior”, “restless sleep”, “dream acting”, “acting out dreams”, “sleep-related violence”, “RBD”, “night terrors”, “sleep interruptions”, “thrashing in bed”, “vivid dreaming”, “dream aggression”, “sleep activity”, “disruptive sleep behavior”, “active dreaming”, “talking in sleep”, “nocturnal activity”, “sleep talking”, “kicking in sleep”, “abnormal sleep behavior”, “aggressive nocturnal behavior”, “nighttime restlessness”, “agitated sleep”],

“Hyposmia”: [“smell impairment”, “reduced smell”, “Hyposmia”, “smelling issues”, “weak sense of smell”, “loss of smell”, “can’t smell”, “faint odors”],

“Asymmetric vague shoulder pain”: [“shoulder pain”, “unequal shoulder pain”, “unexplained shoulder soreness”, “asymmetrical shoulder pain”, “shoulder tenderness”, “intermittent shoulder pain”, “mild shoulder ache”, “one-sided shoulder discomfort”, “vague pain in shoulder”, “shoulder pain without cause”, “shoulder pain without injury”, “shoulder pain on one side”, “shoulder soreness”, “shoulder pain without explanation”, “uneven shoulder ache”, “Asymmetric vague shoulder pain”, “one-sided shoulder pain”, “one shoulder pain”, “shoulder discomfort”, “occasional shoulder ache”, “shoulder stiffness”, “random shoulder pain”, “shoulder pain that comes and goes”, “irregular shoulder pain”, “shoulder hurts”, “shoulder ache”],

“Depression”: [“feeling down”, “low mood”, “depressed”, “hopeless”, “lack of interest”, “persistent sadness”, “melancholy”, “mental”, “emotional numbness”, “feeling empty”, “loss of interest”, “Depression”, “feeling worthless”, “hopelessness”, “sad”, “low spirits”, “lack of pleasure”, “chronic unhappiness”, “feeling blue”, “funk”, “sadness”],

“Impaired color vision”: [“can’t see colors”, “color vision deficiency”, “color blindness”, “dull colors”, “Impaired color vision”, “colors seem faded”, “trouble with colors”],

“Erectile dysfunction”: [“ED”, “trouble with erection”, “Erectile dysfunction”, “sexual dysfunction”, “intimacy issues”, “can’t get hard”, “sexual issues”, “impotence”],

“Reduced arm swing”: [“less arm movement”, “one arm swing”, “arm stiffness”, “arm rigidity”, “arm doesn’t swing”, “Reduced arm swing”, “stiff arm walk”, “arm swing imbalance”],

“Increased stride time variability”: [“change in walk pattern”, “uneven walking”, “gait variability”, “irregular stride”, “stride irregularities”, “walking issues”, “Increased stride time variability”],

“Urinary dysfunction”: [“urination problems”, “urine”, “frequent urination”, “bladder control problems”, “urinary issues”, “bladder dysfunction”, “leaking urine”, “Bladder problems”, “Urinary dysfunction”, “incontinence”, “urinary incontinence”, “bladder control issues”, “bladder issues”, “pee”, “peeing issues”],

“Pain”: [“aching joints”, “tender pain”, “radiating pain”, “debilitating pain”, “excruciating pain”, “muscular discomfort”, “visceral pain”, “pain”, “unrelenting pain”, “tenderness”, “throbbing pain”, “nerve pain”, “cramping”, “ache”, “back pain”, “twinge of pain”, “soreness”, “severe pain”, “dull ache”, “pain management”, “pain treatment”, “hurt”, “pain flare-up”, “nagging pain”, “painful swelling”, “arthritis pain”, “subacute pain”, “body aches”, “mild pain”, “pain therapy”, “piercing pain”, “chronic pain”, “pain relief”, “discomfort”, “burning sensation”, “widespread pain”, “neck pain”, “constant pain”, “moderate pain”, “painful sensation”, “pain control”, “acute pain”, “unbearable pain”, “pain episodes”, “neuropathic pain”, “migraine”, “sharp pain”, “inflammation pain”, “stabbing pain”, “headache”, “breakthrough pain”, “painful symptoms”, “shooting pain”, “muscle pain”, “post-surgical pain”, “stiffness”, “numbness and pain”, “spasms”, “joint pain”],

“Insomnia”: [“wide awake”, “restless sleep”, “insomnia”, “can’t sleep”, “trouble sleeping”, “nighttime awakenings”, “frequent waking”, “sleepless”, “Insomnia”, “Sleep Disturbances”, “sleep problems”, “sleep disorder”, “sleep disruption”, “sleep issues”, “wakeful nights”, “sleep”],

“Anxiety”: [“nervousness”, “stress”, “anxious”, “Anxiety”, “nervous”, “overthinking”, “restlessness”, “anxious feelings”, “worrying”, “tense”],

“Cognitive impairment”: [“forgetfulness”, “memory loss”, “confusion”, “Cognitive impairment”, “cognitive decline”, “difficulty concentrating”, “mental fog”, “brain fog”],

“Fatigue”: [“weary”, “exhaustion”, “knocked out”, “tiredness”, “Fatigue”, “cranky”, “sleepy”, “lack of energy”, “low energy”, “bed”, “exhausted”, “worn out”],

“Bradykinesia”: [“slow to move”, “reduced speed of movement”, “Bradykinesia”, “delayed movements”, “sluggish motion”, “movement slowness”, “slow movement”],

“Rigidity”: [“Rigidity”, “muscular rigidity”, “rigid muscles”, “hard to move”, “stiff muscles”, “inflexible joints”, “muscle stiffness”, “tight muscles”],

“Tremors”: [“shaky movements”, “involuntary shaking”, “Tremors”, “trembling hands”, “hand tremble”, “body shakes”, “shaking”, “tremor”],

“bulbar_dysfunction”: [“bulbar dysfunction”],

“Dysarthria”: [“speech disorder”, “strained voice”, “slow speech”, “talking difficulties”, “hard to verbalize”, “voice changes”, “weak voice”, “speaking fatigue”, “stammering”, “speech impairment”, “unclear speech”, “hard to understand”, “trouble pronouncing”, “difficulty articulating”, “struggling to talk”, “choppy speech”, “slurred speech”, “mumbling”, “garbled speech”, “incoherent speech”, “difficulty speaking”, “stuttering”, “altered speech”, “speech problem”, “hard to speak”, “speech difficulties”, “slurring words”, “nasal speech”, “speaking problems”, “jumbled speech”, “speech changes”],

“Dysphagia”: [“can’t swallow”, “issues with swallowing”, “difficulty eating”, “choking on food”, “chewing problems”, “painful swallowing”, “throat discomfort”, “food getting stuck”, “swallowing discomfort”, “hard to swallow”, “swallowing trouble”, “food sticking in throat”, “difficulty chewing”, “eating difficulties”, “trouble eating”, “food aspiration”, “pain when swallowing”, “swallowing problems”, “hard to eat”, “fear of choking”, “coughing when eating”, “gagging when eating”, “hard swallowing”, “sore throat while eating”, “swallowing pain”, “trouble swallowing”, “difficulty swallowing”, “swallowing disorder”, “feeling of food stuck”, “swallowing difficulty”],

“On-Off Periods”: [“motor fluctuations”, “on periods”, “off periods”, “medication not working”, “variable symptom control”, “early morning off”, “periods of poor mobility”, “drug-induced motor complications”, “unpredictable symptom control”, “medication off time”, “inconsistent medication effect”, “wearing-off phenomenon”, “fluctuating response”, “symptom fluctuations”, “sudden OFF”, “dose failure”, “medication on time”, “peak dose dyskinesia”, “medication wearing off”, “dyskinesia”, “motor symptom variability”, “increased symptoms”, “medication response fluctuations”, “medication cycle fluctuations”, “good ON time”, “deteriorating medication effect”, “levodopa wearing off”, “medication-related mobility changes”, “medication-related motor issues”, “erratic symptom relief”, “levodopa-induced dyskinesia”, “end-of-dose wearing off”, “dose wearing off”, “delayed ON”, “bad OFF time”],

“Postural instability”: [“staggering”, “balance issues”, “leaning”, “difficulty standing upright”, “wobbling”, “swaying”, “unsteady”, “balance problems”, “unsteady standing”, “balance difficulties”, “poor balance”, “dizziness”, “loss of balance”, “instability standing”, “standing issues”, “falling easily”, “balance”, “imbalance”, “difficulty standing”, “Postural instability”],

“Falls”: [“ground”, “falls”, “help”, “serious fall”, “slipped”, “hurt myself falling”, “slipping”, “tumbled”, “frequent falling”, “stumble”, “dropped”, “stumbled”, “sudden fall”, “falling”, “railing”, “plummeted”, “caught myself falling”, “lost balance”, “near fall”, “walking”, “unexpected fall”, “hit the ground”, “tumble down”, “staggered”, “knocked down”, “almost fell”, “shuffle”, “rolled over”, “collapse”, “pitched forward”, “accident”, “leaning”, “shower”, “bruise”, “care”, “fell”, “unsteady”, “hurt”, “lost my balance”, “crashed”, “wiped out”, “floor”, “toppled”, “falling down”, “took a spill”, “imbalance”, “seat”, “surface”, “catch”, “stairs”, “collapsed”, “slip”, “recovery”, “grabbing”, “tripping”, “fall scare”, “stumbling”, “dizziness”, “loss of balance”, “terrain”, “trip”, “injury”, “slid”, “caught”, “face plant”, “fallen”, “toppled over”],

“Gait difficulties”: [“trouble walking”, “walking instability”, “slow gait”, “gait freezing”, “gait asymmetry”, “unpredictability of my legs”, “gait disturbance”, “walking difficulty”, “festinating gait”, “gait difficulties”, “shuffling gait”, “dragging feet”, “abnormal walk”, “irregular gait”, “walking issues”, “freezing of gait”, “mobility issues”, “uneven gait”, “shuffling”, “walking impairment”, “shuffling steps”, “unsteady walk”],

“Assistive Device Use”: [“mobility scooter”, “use of cane”, “supportive devices”, “walking stick”, “adaptive equipment”, “handrails”, “scooter”, “braces”, “orthotic devices”, “rollator”, “assistive walking devices”, “mobility aids”, “grab bars”, “wheelchair use”, “assistive devices”, “using walker”, “adaptive chair”, “walking aids”],

“Freezing of gait”: [“immobilized”, “sudden stop”, “stuck in place”, “freezing episode”, “feet glued”, “gait freeze”, “Freezing of gait”, “freezing”, “can’t lift feet”, “can’t move”, “start hesitation”, “can’t move feet”, “movement hesitation”, “walking freeze”, “sudden stop walking”, “frozen gait”, “legs won’t move”, “can’t step”, “temporary paralysis”, “motor block”, “momentary freeze”],

“Dyskinesia”: [“muscle twitching”, “hyperkinesia”, “motor restlessness”, “unintended muscle movements”, “drug-induced movement disorder”, “involuntary movements”, “fidgeting”, “athetosis”, “abnormal posturing”, “muscular jerks”, “spontaneous movements”, “unpredictable movements”, “levodopa-induced dyskinesia”, “uncontrolled movements”, “writhing movements”, “restless movements”, “muscle rigidity”, “jerky movements”, “chorea”, “dyskinesia”, “jerking movements”, “fluctuating movements”],

“Dystonia”: [“muscular tension”, “muscular spasms”, “abnormal postures”, “abnormal muscle tone”, “neck spasms”, “sustained muscle contractions”, “twisting movements”, “focal dystonia”, “generalized dystonia”, “muscle stiffness”, “writer’s cramp”, “involuntary muscular contractions”, “attack”, “legs wouldn’t work”, “muscle attack”, “abnormal body positions”, “task-specific dystonia”, “sustained postures”, “repetitive movements”, “torticollis”, “twisting postures”, “body distortion”, “muscle rigidity”, “muscle twisting”, “dystonia”, “muscle attacks”, “muscle cramping”, “limb dystonia”],

“Related Disorders”: [“normal pressure hydrocephalus”, “progressive supranuclear palsy”, “parkinsonian gait”, “tardive dyskinesia”, “bradykinesia”, “olivopontocerebellar atrophy”, “ataxia”, “benign essential tremor”, “frontotemporal dementia”, “vascular parkinsonism”, “paraneoplastic syndromes”, “restless legs syndrome”, “essential tremor”, “secondary parkinsonism”, “myoclonus”, “striatonigral degeneration”, “shy-drager syndrome”, “akathisia”, “drd”, “psychogenic movement disorder”, “drug-induced parkinsonism”, “td”, “lewy body dementia”, “spinocerebellar ataxia”, “parkinson-plus syndrome”, “neuroleptic malignant syndrome”, “dystonia”, “multiple system atrophy”, “postural instability”, “parkinsonism”],

“Autonomic Dysfunction”: [“dysautonomia”, “Autonomic Dysfunction”, “temperature regulation problems”, “irregular heartbeat”, “autonomic issues”],

“Orthostatic hypotension”: [“light-headedness on standing”, “postural hypotension”, “Orthostatic hypotension”, “sudden dizziness”, “dizzy standing”, “fainting spells”, “low blood pressure”, “blood pressure issues”],

“Altered Sweating”: [“sweat profusely”, “sweating imbalance”, “sweating fluctuation”, “overactive sweat glands”, “drenching sweats”, “lack of sweat”, “heavy sweating”, “profuse perspiration”, “sweating disorder”, “failed sweat response”, “increased sweating”, “sweating difficulty”, “sweating irregularities”, “sweating dysfunction”, “clammy skin”, “night sweats”, “sweating too much”, “sweaty palms”, “no sweat”, “sweating abnormalities”, “anhidrosis”, “lack of perspiration”, “sweat excessively”, “abnormal perspiration”, “reduced sweating”, “non-sweating”, “excessive sweating”, “sweat attacks”, “difficulty sweating”, “sudden sweating”, “underactive sweat glands”, “sweating disturbance”, “sweating problem”, “heat intolerance”, “uncontrolled sweating”, “hyperhidrosis”, “sweat gland issues”, “excessive perspiration”, “sweating episodes”, “dry skin”],

“Psychosis”: [“paranoid delusions”, “psychotic symptoms”, “unreal perceptions”, “schizophrenia-like symptoms”, “psychosis”, “distorted reality”, “visual hallucinations”, “psychotic depression”, “psychotic break”, “psychotic episode”, “psychotic behavior”, “delusions”, “paranoia”, “bizarre delusions”, “persecutory delusions”, “seeing things”, “irrational thoughts”, “auditory hallucinations”, “psychotic disorder”, “delusional thinking”, “hallucination”, “disorganized thinking”, “false beliefs”, “reality distortion”, “paranoid thinking”, “hearing voices”, “grandiose delusions”, “hallucinations”],

“Mental Health”: [“mental fatigue”, “psychological distress”, “stress”, “emotional strain”, “mental well-being”, “mental strain”, “anxiety”, “psychological well-being”, “emotional problems”, “mental health support”, “mental resilience”, “emotional challenges”, “mental health”, “emotional well-being”, “mental toll”, “mental health issues”, “emotional support”, “depression”, “mental health concerns”, “psychological issues”, “psychological support”, “psychological health”, “emotional distress”, “mental health struggles”, “mental health care”, “emotional health”],

“Cognitive decline”: [“forgetfulness”, “mental confusion”, “short-term memory loss”, “memory decline”, “cognitive slowing”, “memory deterioration”, “cognitive changes”, “cognitive dysfunction”, “mental fuzziness”, “brain fog”, “can’t remember”, “advanced memory issues”, “forgetful”, “memory loss”, “declining memory”, “memory lapses”, “memory issues”, “major forgetfulness”, “slow to respond”, “cognitive deterioration”, “mental decline”, “significant cognitive decline”, “mental slowness”, “brain slowing”, “cognitive difficulties”, “cognitive struggles”, “forgetting things”, “thinking issues”, “cognitive loss”, “cognitive decline in aging”, “cognitive decline”, “sluggish thought”, “cognitive impairment”, “disorientation”, “dementia”, “severe memory loss”, “mental fogging”, “confused”, “mental deterioration”, “losing cognition”, “delayed cognition”, “mental fog”, “thinking delay”],

“addiction issues”: [“excessive sexual behavior”, “addictive tendencies”, “compulsive shopping”, “alcohol abuse”, “substance dependence”, “pathological gambling”, “excessive gambling”, “alcoholism”, “compulsive eating”, “overeating”, “compulsive behavior”, “problem gambling”, “addictive behavior”, “narcotic abuse”, “alcohol addiction”, “impulse control disorder”, “substance abuse”, “addiction problems”, “binge eating”, “drug addiction”, “drug abuse”, “habitual overeating”, “gambling”, “chemical dependency”, “compulsive”, “prescription drug abuse”, “compulsive gambling”, “sexual compulsivity”, “opioid addiction”, “drug dependency”, “internet addiction”, “addictive habits”, “addictive personality”, “sex addiction”],

“Drooling”: [“excessive saliva”, “saliva control problems”, “drool”, “salivating”, “drooling issues”, “sialorrhea”, “dribbling saliva”, “mouth drooling”, “uncontrolled saliva”, “drooling at night”, “saliva management”, “constant drooling”, “spitting”, “saliva accumulation”, “salivary control”, “drooling”, “drooling problems”],

“medication”: [“Eldepryl”, “tolcapone”, “pramipexole”, “Symmetrel”, “medication”, “Cogentin”, “INBRIJA”, “carbodopa”, “Gocovri”, “drug”, “meds”, “Xadago”, “Comtan”, “rasagiline”, “selegiline”, “impax”, “carbidopa”, “Zelapar”, “mao b inhibitor”, “Nourianz”, “azilect”, “Requip”, “Anticholinergics”, “Duopa”, “Ongentys”, “pill”, “Sinemet”, “apokyn”, “entacapone”, “Rytary”, “ropinirole”, “started taking”, “baclofen”, “levodopa”, “Levodopa”, “nuplazid”, “med”, “Dopamine agonists”, “benztropine”, “l-dopa”, “istradefylline”, “safinamide”, “Tasmar”, “rotigotine”, “dopamine”, “opicapone”, “Artane”, “new meds”, “COMT inhibitors”, “Osmolex ER”, “Amantadine”, “Mao-B inhibitors”, “clonazepam”, “trihexyphenidyl”, “Mirapex”, “Neupro”, “levadopa”],

“treatment”: [“disease stage”, “specialist visit”, “therapist”, “lsvt”, “Exercise physiologist”, “speech therapy”, “speech therapist”, “symptom management”, “treatments”, “occupational therapist”, “physician”, “rehab”, “mph”, “speech issues”, “therapy”, “physical therapist”, “treatment side effects”, “holistic approaches”, “treatment”, “neurologist”, “dr”, “healing”, “nursing facility”, “neuro”, “hospital”, “nurse”, “physician’s assistant”, “md”, “symptom”, “therapy sessions”, “doctors”, “doc”, “dpt”, “doctor”, “alternative treatment”, “neurologist appointment”, “treatment options”, “physiotherapy”, “pt”],

“DBS”: [“parkinson’s surgery”, “neurosurgery”, “surgical treatment for Parkinson’s”, “dbs outcomes”, “dbs”, “electrical stimulation brain”, “dbs procedure”, “brain surgery”, “surgical options for Parkinson’s”, “dbs device”, “dbs implant”, “dbs benefits”, “deep brain stimulation”, “brain pacemaker”, “brain stimulation therapy”, “dbs surgery”, “neurological surgery”, “surgery”, “neurosurgical procedure”, “dbs therapy”, “brain stimulation surgery”, “implanting dbs device”, “brain stimulation treatment”, “dbs treatment”, “neurostimulator”, “dbs risks”],

“exercise”: [“basketball”, “elliptical”, “muscle building”, “move”, “fitness class”, “bike”, “bodybuilding”, “tai chi”, “swimming”, “mountain biking”, “water aerobics”, “endurance training”, “badminton”, “running”, “rollerblading”, “parcour”, “parkour”, “circuit training”, “dance”, “tango”, “power walking”, “pilates”, “physical fitness”, “jogging”, “tennis”, “skating”, “yoga”, “football”, “race”, “sprinting”, “5k”, “skateboarding”, “group fitness”, “boxing”, “interval training”, “kettlebell workout”, “cardio workout”, “aerobics”, “surfing”, “skiing”, “marathon training”, “outdoor activities”, “rock climbing”, “spinning”, “workout”, “10k”, “soccer”, “ride”, “walk”, “HIIT”, “moving”, “balance exercises”, “sports”, “kickboxing”, “weight lifting”, “cycling”, “strength training”, “gym”, “zumba”, “rowing”, “aqua aerobics”, “volleyball”, “crossfit”, “barre”, “exercise routine”, “snowboarding”, “spin class”, “calisthenics”, “functional training”, “personal training”, “hiking”, “boot camp”, “trail running”, “stretching”, “mobility exercises”, “exercise”, “bicycling”],

“organizations”: [“wilkins parkinson”, “Parkinson’s foundation symposium”, “parkinson’s foundation”, “brian grant foundation”, “Fox”, “fundraising”, “winning round foundation”, “Foundation”, “Neuro Challenge”, “Michael J. Fox”, “apda”],

“Quality of Life”: [“going natural”, “diet changes”, “illness impact”, “disease milestones”, “disability”, “driving issues”, “disease progression”, “quality of life”, “daily challenges”, “home adjustments”, “living with disease”, “work adjustments”, “day-to-day”, “care routine”, “new normal”, “social isolation”, “recent milestone”],

“community”: [“patient forum”, “community involvement”, “community support”, “support”, “support network”, “moving day”, “peer support”, “support group”, “social support”],

“diagnosis”: [“diagnostic journey”, “Parkinson’s diagnosis”, “late-onset Parkinson’s”, “confirming diagnosis”, “diagnosis”, “yopd”, “diagnosed with Parkinson’s”, “diagnosed”, “early-onset Parkinson’s”],

“caregiver terms”: [“caregiver”, “caregiving challenges”, “caring for spouse”, “caring for parent”, “caregiver support”, “family caregiver”, “spousal caregiver”, “caregiver experience”, “caregiver journey”],

“research”: [“trial”, “research study”, “clinical study”, “research updates”, “medical research”, “medical trial”, “clinical trial”, “Parkinson’s research”, “research breakthroughs”],

“advocacy”: [“health advocacy”, “raising awareness”, “advocacy efforts”, “advocating for patients”, “community advocacy”, “disease awareness”, “Parkinson’s awareness”, “patient advocacy”],

“Other”: [“cure”, “help”, “fight against Parkinson’s”, “support dog”, “overcoming challenges”, “living with handicap”, “illness”, “handicap”, “challenge”, “adapting to illness”, “fight”, “helping”, “travel concerns”, “support animal”, “disease”, “personal battle”],

“parkinson”: [“parkies”, “parkinson”, “parkie”, “pd”, “pwp”],

“gene”: [“PARK1”, “VPS35”, “SNCA”, “DJ1”, “PARK2”, “PARK7”, “PARK17”, “GBA”, “lrrk2”, “PARK6”, “PINK1”, “PRKN”, “PARK8”]}

## Appendix B

The optimized hyperparameters for each classifier were as follows:

- Naïve Bayes: α = 0.1, class_prior = None, fit_prior = True, force_alpha = True;
- Random Forest: 100 estimators, entropy criterion, max features = sqrt, min_samples_leaf = 2, random_state = 42;
- XGBoost: binary logistic objective, learning_rate = 0.1, max_depth = 3, subsample = 0.8, colsample_bytree = 0.8, n_estimators = 100, tree_method = hist, random_state = 42;
- Decision Tree: Gini criterion, max_depth = 30, min_samples_leaf = 2, splitter = best, random_state = 42;
- SVM: linear kernel, C = 1, probability = True, decision_function_shape = ovr;
- AdaBoost: SAMME algorithm, 100 estimators, learning_rate = 1, random_state = 42
- KNN: Euclidean metric, n_neighbors = 8, weights = uniform;
- A soft-voting ensemble classifier was implemented, combining KNN, SVM, Random Forest, AdaBoost, Naïve Bayes, Decision Tree, and XGBoost models.
